# Ureteroscopic management of upper tract transitional cell carcinoma and ureteropelvic obstruction

**DOI:** 10.4103/0970-1591.44262

**Published:** 2008

**Authors:** Sebastien Crouzet, Andre Berger, Manoj Monga, Mihir Desai

**Affiliations:** Glickman Urological and Kidney Institute, The Cleveland Clinic Foundation, Cleveland, Ohio and Department of Urology, University of Minnesota, USA

**Keywords:** Upper tract transitional cell carcinoma, ureteropelvic obstruction, ureteroscopy

## Abstract

**Introduction:**

Technological advances have increased the application of the endoscopic management of upper tract transitional cell carcinoma (TCC) and ureteropelvic junction obstruction (UPJO).

**Materials and Methods:**

Published, peer-reviewed articles on endoscopic treatment of upper tract TCC and UPJO were identified using the MEDLINE database.

**Results:**

Although nephroureterectomy remains the gold standard for upper tract TCC treatment, low-grade, low-stage and small tumors, especially in patients with solitary kidneys or poor renal function can be managed with encouraging success rates, despite the considerable recurrence rate. Endoscopic alternatives to pyeloplasty for UPJO can be used especially in cases with absence of crossing vessels, stricture length less than 1.5 cm, severe hydronephrosis and renal function less than 30%.

**Conclusion:**

Proper patient selection is critical for the successful endoscopic management of treatment of upper tract TCC and UPJO.

## INTRODUCTION

Endourology has undergone a dramatic evolution over the last few decades. This evolution has been driven by improvements in flexible ureteroscope size, active deflection and image quality, advances in videoimaging, diversification of small disposable instrumentation and availability of efficient energy delivery intracorporeally mainly via the Holmium:YAG laser. This article reviews the current status, technical nuances and patient selection of the endoscopic treatment of upper tract transitional cell carcinoma (UTTCC) and ureteropelvic junction obstruction (UPJO).

## TRANSITIONAL CELL CARCINOMA

Primary urothelial carcinoma of the upper tract accounts for approximately 5% of all urothelial tumors.[1] It has a propensity for multifocality, local recurrence and development of metastasic disease, especially with high-grade lesions.[2]

Upper tract TCC treatment is primarily guided by the stage and grade of tumor. In recent years a number of investigators have reported results of endoscopic treatment of upper urinary tract tumors based on efforts to preserve renal function in patients with single kidneys or bilateral tumors.

### Diagnosis - imaging and biopsy

Endoscopic inspection and biopsy are essential to ensure an accurate diagnosis of upper tract TCC. Flexible ureteroscopes allow reliable assessment of the entire collecting system.[3]

### Urinary cytology

The sensitivity of urinary cytology for UTTCC as a whole has been shown as poor in many studies with detection rates as low as 29%.[4] However, cytological detection rates for G3 tumors or carcinoma-*in-situ* approach 100%.[5]

### The role of biopsy

Endoscopic sampling combined with cytopathologic techniques can permit positive diagnosis and accurate grading of upper tract TCC.[6] Whilst simple inspection of upper tract lesions has been shown to accurately predict low- and high-grade TCC in 71% and 80% of cases respectively,[7] biopsy of the suspect lesion within the upper tract leads to a formal diagnosis and information regarding stage and grade.

### Diagnostic imaging

Many departments still currently use a single imaging modality in the hematuria clinic setting. For solid renal masses smaller than 3 cm on ultrasound (US) has been shown to be more sensitive than excretion urography (IVU).[8]

Multiphase spiral computerized tomography (CT) to demonstrate renal pelvic and ureteric anatomy has also been compared to IVU with intrarenal and mid-ureteric results favoring CT.[9] Magnetic resonance urography (MRU) using gadolinium labelled contrast media is yet to find a role in the initial diagnosis of UTTCC.[10]

Virtual ureterorenoscopy is a promising technology, however, remains limited due to difficulties with spatial resolution and the inability to detect subtle changes of mucosal appearance suggestive of Carcinoma *in situ* (CIS).[11]

### Staging

Imaging of the upper urinary tract uncommonly provides accurate staging information. The use of CT is standard in the assessment of the local and distant stage of UTTCC. Extensive local invasion on CT usually corresponds with histological findings and carries a grim prognosis. MRI has been shown to be insensitive in local staging.[12]

Endoluminal ultrasound may hold some promise in assessing invasion.[13]

### Patient selection

For patients with a normal contralateral kidney and normal renal function, the standard of care remains a nephroureterectomy. Inability to completely resect upper tract tumors may occur in up to 32% of patients. Location of tumor does not impact the initial tumor-free rate or recurrence rate. However, patients with tumors larger than 1.5 cm have only a 36% likelihood of being rendered tumor-free compared to 91% for tumors smaller than 1.5 cm. Patients with multifocal disease are also more likely (50%) to have incomplete resections.[14]

Recurrence of transitional cell cancer following electrocautery fulguration, resection or laser ablation may occur in the renal pelvis (37%), ureter (43%) or bladder (41%) at three-year follow-up.[15] The rate of ureteral perforation and stricture has been reported to be 10% and 9% respectively.[15]

The local recurrence rate following laser resection of upper tract tumors through the flexible ureteroscope has been reported to occur in 33% of cases over 3-132 months follow-up.[16] Grade 2 tumors are more likely (44%) to recur than Grade 1 tumors (26%). In addition, tumors larger than 1.5 cm are more likely to recur (50%) than tumors smaller than 1.5 cm (25%); however, this in part may be due to incomplete resection.[14]

Stricture formation may be less likely to occur with Nd:YAG tumor ablation as compared to electrosurgical resection.[17] If a malignant stricture is diagnosed, nephroureterectomy is recommended.

Long-term endoscopic surveillance, with flexible cystoscopy every three months and flexible ureteroscopy every six months for the first two years, followed by cystoscopy every six months and annual flexible ureteroscopy is imperative to ensure early detection and therapy for recurrences.[15] In a review of 199 patients, only 7% undergoing ureteroscopic treatment of transitional cell cancer progressed to nephroureterectomy over 3-132 months of follow-up.[16]

Ureteroscopic treatment of upper tract tumors is not considered adequate therapy for patients with high-grade or invasive lesions. In addition, the increased rate of residual tumor and tumor recurrence with tumors >1.5 cm suggests that these larger tumors are perhaps better managed by a percutaneous approach.[14] In a recent study, 83 patients (93% Ta and 90% Grade 1 or 2), median follow-up of 4.6 years, underwent endoscopic management for UTTCC. Upper tract recurrence was reported in 55% of the patients and and bladder recurrence in 45%. Nephroureterectomy was required in 33% of the patients. Non Ta stage and high grade correlated with cancer death.[18] The key patient selection criteria are a tumor less than 1.5cm, unifocal, low-grade, and superficial. These four criteria offer the greatest predictors of the success of ureteroscopic treatment.[19]

### Retrograde ureteroscopic treatment of transitional cell carcinoma

Compared to percutaneous treatment, ureteroscopy has the advantage of preserving the integrity of the urinary tract.

For tumors in the distal to mid-ureter semi-rigid ureteroscope should be used. For upper ureter and intrarenal lesions a flexible ureteroscope is the instrument of choice. It has been recommended to minimize high-pressure irrigation during these procedures, due to concerns that high intrarenal pressures may promote pyelovenous or pyelolymphatic migration of malignant cells.[20] However, to date, ureteroscopy and ureteroscopic resection have not been associated with a higher risk of development of metastatic disease.[21]

For diagnosis and surveillance it is important to minimize false interpretations due to inadvertent iatrogenic ureteral trauma with a guide-wire. A gentle retrograde pyelogram with a semi-rigid ureteroscope is performed to place a double-floppy tip guide-wire to the mid-ureter. Contrast is injected through the scope to obtain a fluoroscopic “map” of the collecting system.

For tumor ablation a retrograde pyelography should be performed at the outset of the procedure. The placement of a safety guide-wire at the outset of the procedure is crucial prior to biopsy or resection of a lesion.

Following a ureteroscopic biopsy, it is important to remove the basket or forceps along with the ureteroscope to avoid any loss of tissue within the working channel. The specimens should be hand-delivered fresh in saline. Specimens may also be obtained by aspiration or saline wash before or after tumor ablation.[14,22] Using these techniques, the accuracy of grading upper tract tumors can be as high as 97%.[23]

Currently the Holmium YAG laser (1.0 J, 10 Hz) is the most commonly used energy to ablate upper tract tumors ureteroscopically. Alternatively, 3F electrode or Nd:YAG laser (15-30 W, 2 sec) can be used to treat the base of the tumor. However, it should be noted that most of the series reporting long-term follow-up of ureteroscopic management of upper tract tumors have used non-laser modalities, and it remains to be determined whether recurrence rates will be similar.

A systematic evaluation of the remaining collecting system is performed to exclude synchronous multifocal disease. Staged resection at four to eight-week intervals may be indicated for large tumors or if visibility is obscured by bleeding or clot.[15] A ureteral stent is left indwelling for 3-14 days depending on the extent of resection. If a ureteral perforation is noted, the procedure should be terminated and a ureteral stent placed over the safety wire; in this case, the stent is usually left in place for four to six weeks to allow for complete healing of the ureter.[15]

Patients should undergo surveillance cysto-ureteroscopy and cytology on a quarterly basis and flexible ureteroscopy on a semiannual basis for the first two years after resection. Radiographic follow-up of the treated collecting system as a “stand alone” study is inadequate, as up to 75% of tumor recurrences are identified only endoscopically.[14] Imaging of the contralateral kidney is usually performed on an annual basis due to the real, albeit low (< 5%), risk of developing contralateral disease.

## URETEROPELVIC JUNCTION OBSTRUCTION

Options for surgical management of ureteropelvic junction obstruction (UPJO) now include the following: balloon dilation; antegrade, retrograde or cutting balloon endopyelotomy; and open or laparoscopic pyeloplasty. The minimally invasive endoscopic options have become widely accepted because of the reduced morbidity, operative time and hospital stay, despite inferior results compared to open pyeloplasty.[24] Laparoscopic pyeloplasty is now recognized as having equivalent results to the open procedure with less post operative morbidity but longer operative times.[25]

The retrograde ureteroscopic incisional approach was first reported in 1986.[26] Since its first reported series,[27] many refinements have been made to improve the technique, safety and results.

### Preoperative imaging

The diagnostic symptoms of UPJO are hydronephrosis and obstruction. Hydronephrosis may be demonstrated by various scanning methods including CT, renal US, MRI, and IUV. MAG-3 diuretic renography is helpful to delineate the relative function of the kidney, the type of drainage curve, and the output efficiency.

Imaging also defines the etiology and anatomy of the patient. IVU may demonstrate the location and length of narrowing of the ureter as well as site of insertion and other anatomic considerations. Other causes for hydronephrosis and related pain may be detected such as stones, transitional cell cancer, extrinsic compression or strictures. Congenital abnormalities such as reflux can also lead to such pain. All of these can be ruled out using CT, IVU, or other imaging.

Once the diagnosis is made, imaging allows the selection of the most appropriate treatment. The success of endopyelotomy varies with several factors including relative renal function, the presence of crossing vessels, and the degree of hydronephrosis. The wide variation in anatomy means imaging is crucial. Studies suggest that the most important risk factor for failure of an endopyelotomy procedure is the presence of a crossing vessel.[24,28] Imaging techniques used to identify crossing vessels include conventional angiography, helical CT, contrast-enhanced color Doppler imaging, endoluminal ultrasound and MRI.[29] Whilst helical CT has the advantage of being less invasive and more cost-effective than angiography, it is not as accurate.[30] Endoluminal ultrasound has been shown to be more sensitive than CT at detecting crossing vessels,[32] as well as providing additional anatomical information that may assist in directing the endopyelotomy incision, but has the drawback of providing this information only at the time of the proposed endopyelotomy. Improvements in the quality of CT scanning have increased the accuracy of CT angiography, although it is still not as sensitive as endoluminal ultrasound.[31] We now carry out spiral CT as an initial screening test for the presence of crossing vessels when an endopyelotomy is being considered. We reserve endoluminal ultrasound for use immediately prior to endopyelotomy so as to exclude crossing vessels not identified by CT.

### Patient selection

Several factors including stricture length, presence of crossing vessels, severe hydronephrosis, poor renal function, and previously failed endopyelotomy have been suggested to predict a poor outcome after endopyelotomy.

#### Split Renal Function less than 25% and Massive Hydronephrosis

The degree of hydronephrosis and relative renal function also appear to impact the success after endopyelotomy. Danuser *et al*. reported a decreasing success rate after endopyelotomy with increasing hydronephrosis. They reported a success rate of 87% in patients with a pyelocalyceal volume < 50 cc, which decreased to 81% and 69% with pyelocalyceal volume of 50-100cc and > 100 cc, respectively. Gupta *et al*. reported a decrease in success rate from 92% to 54% in patients with poor renal function.[32]

#### Crossing vessels

The impact of crossing vessels on the outcome of endopyelotomy remains an area of controversy. Several studies have suggested a lower success rate with endopyelotomy in the presence of a crossing vessel at the UPJ. Van Cangh *et al*. reported a success rate of 42% in patients undergoing endopyelotomy in the presence of a crossing vessel compared to 86% in whom a crossing vessel was absent.[24] Similarly, Nakada *et al*. reported a decreased success rate (96% to 64%) of Acucise^®^ endopyelotomy in patients with a crossing vessel.[33] Additionally, crossing vessels are frequently found on exploration in patients requiring a pyeloplasty after a failed endopyelotomy. Knudsen and colleagues reported an 83% incidence of crossing vessels during exploration after failed endopyelotomy.[34] This contrasts with the experience of Gupta *et al*. who attributed the effects of crossing vessels to 4% of endopyelotomy failures in a series of 401 patients.[32]

### Technique

The choice between the antegrade versus ureteroscopic approach for endopyelotomy is made on the basis of surgeon preference, presence of concomitant calculi, and anatomic factors. Overall, the ureteroscopic approach may be preferred because of the reduced morbidity, lack of incision, and the ability to perform as an outpatient procedure compared to the percutaneous approach. The percutaneous approach may be preferred in patients with concomitant calculi that may be treated simultaneously. Additionally, the percutaneous approach may be employed if the body habitus or anatomic factors preclude optimal access to the UPJ. However, availability of miniature and flexible ureteroscopes has reduced the role of anatomic factors in limiting the technical feasibility of ureteroscopic endopyelotomy.

### Ureteroscopic method

A Glidewire (Boston Scientific) is used to cannulate the ureteral orifice under cystoscopic guidance and advanced up the ureter under fluoroscopic guidance until it is noted to coil in the renal pelvis. During this part of the procedure, it may be helpful to inject contrast through a catheter passed over the guidewire in order to delineate the exact location and length of the stricture. The key factor here is to not false pass the area of the stricture.

Normal saline should be utilized for all ureteroscopic procedures. If electrosurgical current is needed during a procedure, the irrigant is converted to sorbitol.

We utilize pressure irrigation for maintaining adequate flow for optimal visualization.

The semi-rigid ureteroscope is advanced alongside the guidewire to dilate the ureteral orifice and evaluate the distal and mid-ureter for unanticipated pathology. If there is difficulty passing the semi-rigid scope an Amplatz superstiff wire (Boston Scientific) can be placed through the working channel of the ureteroscope and the ureteroscope advanced over the wire. In women, the semi-rigid ureteroscope will often reach the UPJ and be adequate to perform an endopyelotomy. Otherwise, a superstiff is advanced through the scope until it is noted to coil fluoroscopically in the renal pelvis and flexible ureteroscope is advanced in the ureter.

In the last decade, it was commonplace to have to dilate the ureter prior to passage of the larger flexible ureteroscopes. However, the introduction of small diameter (i.e. < 9F) actively deflectable flexible ureteroscopes has facilitated ureteral insertion, precluding the need for dilation of the ureteral orifice or tunnel with balloons or shear dilators. Dilation of the intramural ureter beyond 10F for insertion of the new ureteroscopes is required in only 12% of cases.[35] Indeed, passage of a 10F introducer catheter (e.g. 8/10F Amplatz set) for the placement of a safety guidewire is usually sufficient to also smooth the way for passage of the smaller flexible ureteroscopes. The need for balloon dilation is exceedingly rare.

Entering the ureteral orifice by passing the flexible ureteroscope alongside a guidewire in the ureter can be a time-consuming undertaking. The traditional method for inserting the flexible ureteroscope has been to first pass a second floppy guidewire up the ureter and then proceed to backload the ureteroscope over the wire and advance it up the ureter under fluoroscopic guidance. However, guidewire trauma to the working channel of the ureteroscope may shorten the lifespan of these fragile instruments, especially if one tries to backload the ureteroscope on a superstiff guidewire. The recent development of guidewires with a floppy tip on either end has greatly increased the safety margin of this maneuver (Sensorwire, Microvasive Inc., Natick MA). The flexible ureteroscope (FURS) therefore would now be advanced over the Sensor wire, leaving the Amplatz superstiff as a safety wire.

If the FURS is advanced over a wire, rotating the scope to realign the tip such that the guidewire in the working channel is in a 12-o'clock position to “lift” the orifice open may be of benefit. Any forceful effort to advance the ureteroscope up the ureter usually results in buckling of the midshaft of the ureteroscope into the bladder with attendant decrease in the range of tip deflection. Alternatively, one could place a ureteral access sheath (Cook Flexor, 12/14Fr) over the superstiff to establish access for insertion of the flexible ureteroscope, leaving the Sensor wire as the safety wire.

If the endoscope cannot be passed through the area of the stricture, it is helpful then to use a 4 or 5-mm balloon to dilate the area of the stricture so it can be easily traversed with the ureteroscope. A 365-micron laser fiber is inserted orienting the fiber laterally and the stricture is ablated as the ureteroscope is slowly withdrawn. Usually 1.0 J at 10 Hz is sufficient to accomplish the laser incision. This process is repeated until thin fibers are identified. Alternatively, a Hulbert pencil point electrode or 2F and 3F electrosurgical probes may be utilized, using 45 W of pure cut.[36] Care should be taken to incise laterally at the UPJ. The ureteroscope is then removed. Ballon dilation is performed with a 5 or 6mm Uromax Balloon at 20 psi for 5 min. If a waist in the balloon persists, re-incise with the laser fiber. An 8Fr ureteral stent is left indwelling for two to three weeks and urethral catheter left for 24-48 h.

## CONCLUSION

Proper patient selection is the key to successful endoscopic treatment for UTTCC and UPJO. It may be the preferred treatment in patients unfit for major surgery, with bilateral disease or a solitary kidney. Low-grade, low-stage disease of the distal ureter also appears to be particularly well suited to this approach. Patients with superficial tumors less than 1.5 cm in size, unifocal, and low-grade are the best candidates for endourologic treatment. For UPJO, without crossing vessels, stricture length greater than 1.5 cm, severe hydronephrosis and renal function less than 30%, endopyelotomy may be an appropriate surgical option for the patient. Ureteroscopic approach may be preferred because of the reduced morbidity, lack of incision, and the ability to perform as an outpatient procedure compared to the percutaneous approach especially after the development of the modern ureteroscopes.
